# Prevalence, Indications, and Complications of Conversional Surgery After Vertical Banded Gastroplasty: A MBSAQIP Analysis

**DOI:** 10.1007/s11695-024-07353-8

**Published:** 2024-06-11

**Authors:** Juan S. Barajas-Gamboa, Valentina Duran, Gustavo Romero-Velez, Valentin Mocanu, Yung Lee, Ricard Corcelles, Matthew Allemang, Andrew T. Strong, Salvador Navarrete, John Rodriguez, Matthew Kroh, Jerry T. Dang

**Affiliations:** 1 Digestive Disease Institute, Cleveland Clinic Abu Dhabi, Abu Dhabi, United Arab Emirates; 2 Experimental Surgery and Simulation Center, Catholic University of Chile, Santiago, Chile; 3 Digestive Disease Institute, Cleveland Clinic, Cleveland, OH USA; 4 Cleveland Clinic Lerner College of Medicine of Case Western Reserve University, Cleveland, OH USA; 5 Department of Surgery, University of Alberta, Edmonton, AB Canada; 6 Department of Surgery, McMaster University, Hamilton, ON Canada

**Keywords:** Conversional surgery, Revisional surgery, Vertical banded gastroplasty, MBSAQIP

## Abstract

**Purpose:**

Vertical banded gastroplasty (VBG) was once the most popular bariatric procedure in the 1980’s, with many patients subsequently requiring conversional surgery. However, knowledge regarding the prevalence and outcomes of these procedures remains limited. This study aims to determine the prevalence, indications, rate of 30-day serious complications, and mortality of conversional surgery after VBG.

**Materials and Methods:**

A retrospective analysis of the MBSAQIP database from 2020 to 2022 was conducted. Individuals undergoing conversional or revisional surgery after VBG were included. The primary outcomes were 30-day serious complications and mortality.

**Results:**

Of 716 VBG conversions, the common procedures included 660 (92.1%) Roux-en-Y gastric bypass (RYGB) and 56 (7.9%) sleeve gastrectomy (SG). The main indication for conversion was weight gain for RYGB (31.0%) and for SG (41.0%). RYGB had longer operative times than SG (223.7 vs 130.5 min, *p* < 0.001). Although not statistically significant, serious complications were higher after RYGB (14.7% vs 8.9%, *p* = 0.2). Leak rates were higher after SG (5.4 vs 3.5%) but this was not statistically significant (*p* = 0.4). Mortality was similar between RYGB and SG (1.2 vs 1.8%, *p* = 0.7). Multivariable regression showed higher body mass index, longer operative time, previous cardiac surgery and black race were independently associated with serious complications. Conversion to RYGB was not predictive of serious complications compared to SG (OR 0.96, 95%CI 0.34–2.67, *p* = 0.9).

**Conclusions:**

Conversional surgery after VBG is uncommon, and the rate of complications and mortality remains high. Patients should be thoroughly evaluated and informed about these risks before undergoing conversion from VBG.

## Introduction

Vertical banded gastroplasty (VBG) was introduced in the early 1980s as a surgical weight loss intervention and quickly became the most popular bariatric procedure [1]. Pioneered by Dr. Edward E. Mason at the University of Iowa, this technique was developed as an alternative to the jejunoileal bypass surgery, aiming to minimize complications by preserving gastrointestinal anatomy [2–4]. The procedure involved reducing gastric volume to limit food intake, with the band designed to delay the transition of food to the intestines, thereby promoting a sensation of satiety [1–3]. However, it was largely abandoned due to poor long-term weight loss outcomes and complications related to the band, including stomal stenosis, ulceration, and gastroesophageal reflux disease (GERD) [4, 5].

Concurrently, other bariatric surgery procedures such as Roux-en-Y gastric bypass (RYGB) and, later, sleeve gastrectomy (SG) began to gain popularity. These procedures demonstrated better long-term weight loss outcomes and lower complication rates compared to VBG [6–9]. The evolution of laparoscopic techniques in the 1990s and early 2000s further transformed these surgeries into less invasive procedures, resulting in quicker recovery times and reduced complication rates. Consequently, by the latter part of the 2000s and into the 2010s, the adoption of VBG notably declined in favor of these contemporary approaches [10–13].

Nevertheless, despite this transition, there remain patients with VBG anatomy today who may benefit from revisions or conversions to other bariatric procedures, either for weight-related concerns or potential postoperative complications [6, 10, 14–21].

However, current evidence evaluating outcomes of conversional surgery after VGB is limited to retrospective studies or single-center experiences. As revisional procedures become more prevalent, there is an increasing need to evaluate the safety of converting from VGB on a broader scope, using national databases such as the Metabolic and Bariatric Surgery Accreditation and Quality Improvement Program (MBSAQIP).

We hypothesize that conversional surgery from VBG has a high rate of 30-day serious complications due to increased complexity [20, 22–26]. Therefore, the objective of this study was to determine the prevalence, indications, rate of 30-day serious complications, and mortality of conversional surgery after VBG.

## Methods

### Study Design and Population

This is a retrospective cohort study of prospectively collected data. This study included laparoscopic and robotic procedures from patients who underwent conversional procedures from VBG to another bariatric procedure. The primary outcomes of this study were to evaluate the 30-day rate of serious complications and mortality. Serious complication was defined as a composite variable for a patient experiencing one or more of the following within 30 days of surgery: anastomotic leak, postoperative bleeding, reoperation, non-operative intervention, cardiac arrest, MI, cardiopulmonary resuscitation, pneumonia, unplanned intubation, acute kidney injury, VTE, deep surgical site infection, sepsis or cerebrovascular accident.

The secondary outcomes were to identify and characterize indications for conversional procedures after VBG. Patients were included if they were labelled with “Vertical banded gastroplasty” for the variable “Conversions—Previous Metabolic and Bariatric Procedure.”

### Ethical Approvals

This study was reviewed and deemed exempt by the Institutional Review Board (IRB) due to the anonymity of the data.

### Data Source

The Metabolic and Bariatric Surgery Accreditation and Quality Improvement Program (MBSAQIP) database registry was reviewed and analyzed. Only data from 2020 to 2022 was included as there was a modification in 2020 that included additional details on revisional surgery that were previously not reported.

### Patient Variables

Basic demographic data including age, sex, race, and body mass index (BMI) were collected. Patient comorbidities included the following diseases: diabetes, hypertension, GERD, chronic obstructive pulmonary disease (COPD), hyperlipidemia, chronic steroid use, chronic kidney disease, dialysis dependency, venous stasis, preoperative therapeutic anticoagulant use, and obstructive sleep apnea (OSA). Patient history included previous venous thromboembolism (VTE), myocardial infarction (MI), percutaneous coronary intervention, and major cardiac surgery. Functional status variables comprised preoperative functional status (defined as independent, partially dependent, or fully dependent) and American Society of anesthesiologists (ASA) Physical Status classification.

### Statistical Analysis

The descriptive categorical data were expressed as percentages, and the continuous data as mean and standard deviation (SD). Non-parametric continuous data was expressed using median and interquartile range (IQR). Univariate analyses were used to determine baseline differences between groups, using chi-squared tests for categorical data and independent sample t-tests for continuous data.

A multivariable logistic regression analysis was used to identify predictive factors for serious complications and mortality within 30 days. The available case method addressed missing data as all variables had less than 5% missingness. Patient factors and operative time were included in the model. Any variable with a *p*-value < 0.05 in univariate analysis was included in multivariable analysis. Variables were checked for multicollinearity via the variable inflation factors method. The area under the receiver operating characteristic (AUROC) curve and Brier score were used to assess the validity and calibration of the multivariable model. All statistical analyses were completed using STATA 17 statistical software (StataCorp, College Station, TX, USA).

## Results

### Patient Demographics

In this study, we included 740 patients who underwent conversional bariatric surgery after VBG. Of these, 660 (89.2%) patients underwent conversion to RYGB, 56 (7.6%) patients underwent conversion to SG, 11 (1.5%) patients underwent conversion to other procedures, 6 (0.8%) patients underwent conversion to one anastomosis gastric bypass (OAGB), and 7 (0.9%) patients underwent conversion to biliopancreatic diversion with duodenal switch (BPD-DS). Our primary analysis specifically focused on the SG and RYGB subgroups, as they represented the predominant percentage of VBG conversions. However, an additional sub-group analysis for OAGB and BPD-DS was also included. Within this cohort of 716 patients, most of them were female (91.1%). The mean BMI for patients undergoing RYGB was 43.1 ± 9.5 kg/m^2^ and for those undergoing SG was 45.4 ± 10.3 kg/m^2^.

The majority of patients, 580 (81.0%) were classified as American Society of Anesthesiologists (ASA) class 3, indicating severe systemic disease. Prior to surgery, a substantial number of patients, 705 (98.6%) maintained functional independence. Regarding comorbidities and health risk factors, GERD was present in 420 (58.4%) of patients, hypertension in 131 (57.9%), and OSA in 224 (31.2%). Cardiovascular risk profiles included a 8.2% prevalence of VTE, 0.9% incidence of previous major cardiac surgery, and 5.3% history of myocardial infarction.

A minority of the cohort, 14% patients were on long-term steroid therapy, while chronic obstructive pulmonary disease (COPD) was present in 4.7% patients. Chronic kidney disease was noted in 2.9% patients, with a smaller subset of 0.2% patient requiring dialysis, as detailed in (Table 1).

**Table 1:** Patient characteristics

	RYGB *n* = 660	SG *n* = 56	*p-value*
Age, years mean ± SD	57.2 ± 9.3	56.4 ± 8.9	0.003
Female	601 (91.1)	51 (91.1)	0.998
Race White Black Other	488 (73.9) 96 (14.6) 76 (11.5)	43 (76.8) 8 (14.3) 5 (8.9)	0.834
Body mass index (kg/m^**2**^) mean ± SD	43.1 ± 9.5	45.5 ± 10.3	0.076
Functional Status Independent Partially dependent Totally dependent	650 (98.5) 9 (1.4) 1 (0.2)	55 (100) 0 (0.0) 0 (0.0)	0.655
American Society of Anesthesiologists class 1–2 3 4–5	91 (13.8) 534 (80.9) 35 (5.3)	8 (14.3) 46 (82.1) 2 (3.6)	0.853
Smoker	30 (4.6)	5 (8.9)	0.144
Diabetes None or diet-controlled Non-insulin-dependent Insulin-dependent	518 (78.5) 100 (15.2) 42 (6.4)	45 (80.4) 6 (10.7) 5 (8.9)	0.541
Hypertension	123 (59.1)	8 (44.4)	0.226
Gastroesophageal reflux disease	395 (59.8)	25 (44.6)	0.027
Chronic obstructive pulmonary disease	19 (2.9)	1 (1.8)	0.634
Hyperlipidemia	245 (37.1)	15 (26.8)	0.123
Chronic steroids	6 (2.9)	2 (11.1)	0.070
Chronic kidney disease	7 (1.1)	1 (1.8)	0.620
Dialysis dependent	1 (0.2)	0 (0.0)	0.771
History of venous thromboembolism	42 (6.4)	1 (1.8)	0.166
Venous stasis	9 (1.4)	1 (1.8)	0.796
Preoperative therapeutic anticoagulant use	48 (7.3)	3 (5.4)	0.593
Obstructive sleep apnea	203 (30.8)	21 (37.5)	0.296
History of myocardial infarction	11 (1.7)	2 (3.6)	0.305
Previous percutaneous coronary intervention	19 (2.9)	5 (8.9)	0.016
Previous major cardiac surgery	6 (0.9)	0 (0.0)	0.474

Abbreviations: RYGB, Roux-en-Y gastric bypass; SG, sleeve gastrectomy

### Conversion Indications

The most common indications for conversion surgery differed between the two groups. In the RYGB group, the primary indications were weight gain in 205 (31.0%), GERD in 154 (23.3%), inadequate weight loss in 115 (17.4%), dysphagia in 45 (6.8%), and gastrointestinal tract stricture or obstruction in 41 (6.2%) patients. For the SG group, weight gain in 23 (41.0%) and inadequate weight loss in 13 (23.2%) were the most frequent reasons for conversion, followed by GERD in 10 (17.9%), gastrointestinal tract stricture or obstruction in 4 (7.1%), and dysphagia in 1 (1.7%) patient. Detailed indications are summarised in Table 2.

**Table 2:** Indications for surgery and intraoperative findings

	Roux-en-Y gastric bypass *n* = 660	Sleeve gastrectomy *n* = 56	*p-value*
Indication for surgery			
Weight gain	205 (31.0)	23 (41.1)	
Gastroesophageal reflux disease	154 (23.3)	10 (17.9)	
Inadequate weight loss	115 (17.4)	13 (23.2)	
Dysphagia	45 (6.8)	1 (1.8)	
Adhesions	10 (1.5)	1 (1.8)	
Gastrointestinal tract stricture or obstruction	41 (6.2)	4 (7.1)	
Nausea and/or vomiting	31 (4.7)	0 (0.0)	
Other (not listed)	12 (1.8)	1 (1.8)	
Gastrointestinal tract fistula	14 (2.1)	0 (0.0)	
Mechanical malfunction	10 (1.5)	1 (1.8)	
Patient intolerance	8 (1.2)	1 (1.8)	
Persistent comorbidities	7 (1.0)	0 (0.0)	
Patient non-compliance	1 (0.1)	1 (1.8)	
Fluid, electrolyte, or nutritional depletion	2 (0.3)	0 (0.0)	
Gastrointestinal tract ulcer without perforation	1 (0.1)	0 (0.0)	
Abdominal Pain	4 (0.6)	0 (0.0)	
Surgical approach			
Open	29 (4.4)	1 (1.8)	0.350
Laparoscopic	631 (95.6)	55 (98.2)	
Operative time minutes ± SD	223.7 ± 99.5	130.5 ± 52.0	< 0.001
Length of stay median (interquartile range)	2 (1)	1 (1)	0.274

### Procedural Factors

Comparative analysis of procedural factors demonstrated significant differences between the RYGB and SG groups. RYGB conversions had considerably longer operative times, averaging 223.6 ± 99.5 min compared with 130.5 ± 52.0 min for SG, with the difference being statistically significant (*p* < 0.001). In terms of surgical approach, laparoscopic techniques predominated at 95.8% (*n* = 686), followed by 4.2% (*n* = 30) open surgeries. For the RYGB cohort, 631 (85.3%) procedures were performed laparoscopically, of which 12 (1.6%) were converted to open surgery. Additionally, 29 (3.9%) RYGB procedures were performed as open surgeries from the outset. In the SG cohort, 55 (7.4%) procedures were completed laparoscopically, with 1 (0.1%) open SG performed.

The median length of stay for RYGB conversion was 2 (interquartile range 1) days. In contrast, SG patients experienced a shorter hospital stay, with a median of 1 (interquartile range 1) days, however this difference was not statistically significant (*p* = 0.2).

### Postoperative Complications and Mortality

RYGB patients experienced a higher rate of serious complications at 14.7% (*n* = 97), compared to 8.9% (*n* = 5) for SG patients, though this difference was not statistically significant (*p* = 0.2). Complications for RYGB versus SG conversions were not statistically different but included leakage (3.5% vs. 5.4%, *p* = 0.4), bleeding (3.8% vs. 1.8%, *p* = 0.4), reoperation (7.1% vs. 3.6%, *p* = 0.3), non-operative reintervention (4.2% vs. 1.8%, *p* = 0.3), readmission (11.2% vs. 8.9%, *p* = 0.6), and deep surgical site infections (3.9% vs. 5.4%, *p* = 0.6). There was a 1.2% mortality rate noted after RYGB and 1.8% for SG (*p* = 0.7). Further information is presented in Table 3.

**Table 3:** Postoperative complications

	Roux-en-Y gastric bypass *n* = 660	Sleeve gastrectomy *n* = 56	*p-value*
Leak	23 (3.5)	3 (5.4)	0.472
Bleeding	25 (3.8)	1 (1.8)	0.442
Reoperation	47 (7.1)	2 (3.6)	0.312
Non-operative reintervention	28 (4.2)	1 (1.8)	0.371
Readmission	74 (11.2)	5 (8.9)	0.601
Non-operative intervention	28 (4.2)	1 (1.8)	0.371
Cardiac events	5 (0.8)	1 (1.8)	0.418
Pneumonia	14 (2.1)	0 (0.0)	0.271
Unplanned intubation	7 (1.1)	1 (1.8)	0.620
Acute kidney injury	4 (0.6)	0 (0.0)	0.559
Venous thromboembolism	7 (1.1)	0 (0.0)	0.439
Deep surgical site infection	26 (3.9)	3 (5.4)	0.605
Wound disruption	3 (0.5)	0 (0.0)	0.613
Sepsis	9 (1.4)	1 (1.8)	0.796
Cerebrovascular accident	1 (0.2)	0 (0.0)	0.771
Serious complications	97 (14.7)	5 (8.9)	0.236
Death	8 (1.2)	1 (1.8)	0.711

### Multivariable Logistic Regression

Following adjustment with multivariable logistic regression, four variables were independently predictive of 30-day serious complications: previous cardiac surgery, longer operative times, higher BMI, and Black race (Table 4). Type of conversional procedure (SG vs RYGB) was not predictive of serious complications (OR 0.96, 95%CI 0.34–2.67, *p* = 0.9) after adjusting for age, BMI, comorbidities, functional status and operative time. For 30-day mortality, previous cardiac surgery and higher BMI were predictive of death (Table 5). However, conversional procedure type was not predictive of mortality (OR 1.80, 95%CI 0.19–16.8, *p* = 0.6). The serious complication model had an AUROC of 0.73 and Brier score of 0.11 while the mortality model had a AUROC of 0.91 and Brier score of 0.012.

**Table 4:** Significant risk factors for serious complications on multivariable logistic regression

Risk Factor	Adjusted Odds Ratio*	95% confidence interval	p-value
Conversional procedure SG vs RYGB	0.96	0.34–2.67	0.938
Previous cardiac surgery	9.93	1.79–55.0	0.009*
Longer operative time (per hour)	1.32	1.15–1.50	< 0.001*
Higher body mass index (per 10 kg/m^2^)	1.46	1.16–1.85	0.001*
Race Black Other	2.15 1.89	1.21–3.83 0.92–3.86	0.009* 0.082
Older age (per 10 years)	1.05	0.80–1.37	0.748
Partially dependent functional status	1.96	0.42–9.12	0.393
Chronic kidney disease	3.46	0.75–16.0	0.112
Diabetes Non-insulin-dependent Insulin-dependent	1.40 1.84	0.84–4.03 0.78–2.53	0.128 0.264
Gastroesophageal reflux disease	1.48	0.90–2.44	0.119
Chronic obstructive pulmonary disease	2.87	0.95–8.67	0.063
Therapeutic anticoagulation	1.49	0.70–3.16	0.298

Abbreviations: SG, sleeve gastrectomy; RYGB, Roux-en-Y gastric bypass

^*^Significant factors

**Table 5:** Significant risk factors for mortality on multivariable logistic regression

Risk Factor	Adjusted Odds Ratio	95% confidence interval	p-value
Conversional procedure SG vs RYGB	1.80	0.19–16.8	0.604
Previous cardiac surgery	24.7	1.81–336.7	0.016*
Higher body mass index (per 10 kg/m^2^)	2.20	1.15–4.22	0.017*
Older age (per 10 years)	2.41	0.81–7.19	0.116
Partially dependent functional status	4.11	0.28–59.5	0.299
Hyperlipidemia	2.16	0.47–10.0	0.324
Previous myocardial infarction	3.33	0.24–45.6	0.368
Chronic kidney disease	10.3	0.95–111.0	0.055

Abbreviations: SG, sleeve gastrectomy; RYGB, Roux-en-Y gastric bypass

^*^Significant factors

### Sub-group Analysis for other Procedures (Conversions to BPD-DS and OAGB)

For the BPD-DS subgroup, which comprised 7 (0.9%) patients who underwent conversion to biliopancreatic diversion with duodenal switch, the mean age was 48.6 years (± 8.4), mean BMI was 48.8 kg/m^2^ (± 9.5), and mean operative time was 163.7 min (± 68.8). All procedures were performed laparoscopically. Indications included inadequate weight loss (3 cases) and weight gain (4 cases). There was 1 readmission, no serious complications, and no deaths. In the OAGB subgroup, 6 (0.8%) patients underwent conversion to one anastomosis gastric bypass. The mean age was 54.2 years (± 11.0), mean BMI was 47.2 kg/m^2^ (± 14.1), and mean operative time was 282.3 min (± 132.8). Five procedures were laparoscopic, with one conversion to open. Indications were dysphagia (2 cases), inadequate weight loss (3 cases), and weight gain (1 case). There was 1 leak, 2 reoperations, 2 readmissions, 1 deep surgical site infection, and 2 serious complications. No deaths occurred in this subgroup.

## Discussion

Conversional surgeries following VBG are relatively rare, since VBG is no longer performed. Over a period of three years, 740 cases were reported across 902 centers. RYGB and SG are the predominant conversional procedures after VBG, commonly indicated for weight recurrence, insufficient weight loss, and gastroesophageal reflux disease (GERD). RYGB conversions had a longer operative time and higher, though not statistically significant, complication rates compared to SG. SG appeared to have a higher rate of leaks, but this was also not statistically significant. The study identified multiple predictors of serious postoperative complications with the multivariable logistic regression: longer operative times, higher BMI, previous cardiac surgery, and Black race.

Our results are consistent with existing evidence highlighting the relative safety of RYGB as a conversional surgery following VBG [20, 24]. However, with complication rates as high as 14.7% and mortality at 1.2%, a prudent surgical approach is warranted. The high complication rates could be explained due to the complexity of the conversions and the dense adhesions from the primary procedure. These outcomes suggest that conversions from VBG carry more risk compared to other bariatric procedures. Our mortality findings contrast with some reports that indicate a zero-mortality rate, showing a 1.2% mortality in our cohort [24]. Leading indications for conversional surgery reported in the literature include weight gain (33%), insufficient weight loss (16%), and gastrointestinal tract strictures (7%), which correlate with our findings [22, 23]. The mean pre-revisional BMI in our study group was approximately 43 kg/m^2^, which is in line with or slightly higher than reported in previous studies [19, 20, 24].

While previous studies have found certain factors—such as the type of conversional surgery (SG or RYGB), functional status, diabetes, and the use of therapeutic anticoagulation—to be significant, our analysis did not validate these associations. Our study provides the most comprehensive evaluation of surgical outcomes to date following VBG conversion, potentially informing how surgeons discuss the procedure's pros and cons with their patients [25, 26].

Conversional bariatric surgery following VBG often involves selecting between SG and RYGB, each with different surgical techniques and associated risks. A prevailing debate concerns the feasibility of SG post-VBG, given the challenges of stapling through band-induced fibrotic tissue, which is thought to increase the risk of staple line leaks. Meanwhile, in RYGB conversions, the strategic placement of the gastrojejunal anastomosis to avoid VBG scarring raises concerns about creating an extremely small gastric pouch [26, 27].

The possibility of creating a constricted gastric pouch during RYGB conversion might induce early satiety, but it also raises concerns about nutrient absorption and pouch drainage [28]. Such complex surgical outcomes necessitate those decisions be grounded in thorough patient assessments and an intricate understanding of the effects of previous surgeries. In the context of bariatric revision after VBG, the presence of GERD frequently sways the decision towards RYGB due to its potential benefits in managing this condition [28, 29].

Our data suggest that SG could be a viable option for surgeons to consider during VBG conversions. In our analysis, SG reported a lower, though not statistically significant, rate of serious complications compared to RYGB, indicating a potentially safer profile [30, 31]. This finding is significant, especially considering the prevalent surgical concerns regarding SG after VBG due to the risks associated with stapling through a fibrotic stomach from long-term banding and the presumed increased risk of leaks [32]. Although our study demonstrated a slightly higher rate of leak after SG (5.4 vs 3.5%), this was not statistically significant. However, this lack of statistical significance may have been due to a small sample size in the SG cohort resulting in a type 2 error.

Our study presents unique insights, notably the higher incidence of serious complications in RYGB at 14.7%, compared to 8.9% in SG. These differences may be influenced by the longer duration and increased complexity of RYGB procedures and the higher baseline prevalence of GERD, which presents additional surgical challenges [33, 34]. Additionally, the mean age in the RYGB group was significantly higher (*p* < 0.003). Furthermore, the significant correlation between serious complications, longer operative time, higher BMI, previous cardiac surgery, and Black race could be attributed to the complex interaction of physiological, surgical, and recovery-related challenges. Patients with a higher BMI typically have greater adipose tissue, which complicates surgical procedures by hampering organ visibility and access and are more likely to suffer from obesity-related comorbidities such as diabetes and heart disease that can compromise recovery [35]. Additionally, excess body weight can impair wound healing and immune function, increasing infection risks. Longer operative times increase these risks by exposing patients to extended periods of anesthesia, which can lead to thrombotic events, respiratory difficulties, and a heightened inflammatory response that can prolong recovery. Prolonged surgeries also raise the likelihood of intraoperative complications such as excessive blood loss [36, 37]. Therefore, while longer operative times may contribute to increased risks of complications, the association is not directly correlated with the technique itself. Rather, the longer operative times may lead to other situations that affect the complication rates.

Patients with a history of cardiac surgery are at an increased risk of postoperative complications following bariatric surgery due to factors such as the presence of adhesions and scar tissue, reduced cardiac reserve, increased risk of arrhythmias, exacerbated obesity-related comorbidities, and technical challenges resulting from altered chest cavity anatomy [38]. Finally, we believe that individuals of black race are at increased risk of postoperative complications following bariatric surgery due to disparities in access to healthcare, as documented in previous studies. These disparities are based on existing social determinants of health, including residential geographic location and lack of health insurance, leading to unequal access to preventive care, surgery, in-hospital care, and postoperative follow-up [39].

Our study offers significant insights but is not without limitations. The retrospective design and reliance on a specific data collection method, coupled with possible unaccounted confounding variables, could impact the interpretation of the results. Additionally, the absence of detailed comorbidity data such as liver disease and heart failure, the lack of granularity regarding comorbidity severity, and the wide variation in surgical expertise within the MBSAQIP database may limit the study's comprehensiveness. Moreover, the MBSAQIP database is limited to 30-day outcomes. In addition, there was a small sample of patients converted to SG which may result in type 2 errors when comparing complication rates.

Furthermore, it is important to note that data on long-term weight loss following these revisions is not available in the MBSAQIP database. While we could include data on weight loss up to 30 days post-surgery, its clinical relevance is uncertain. Additionally, information regarding the specific centers where these surgeries were performed and the experience level of individual surgeons is not provided. Therefore, we were unable to investigate whether the surgical center and surgeon experience are predictors of serious complications in our analysis. Importantly, the MBSAQIP registry lacks data regarding the surgical approach for the primary procedure (open, laparoscopic, robotic, etc.), which is significant as older VBGs were performed by laparotomy. Nonetheless, our study is the largest study on VBG conversions to date and contributes valuable knowledge to the field, laying the groundwork for future prospective trials.

## Conclusions

Conversional surgery after VBG is uncommon, and the rate of complications and mortality remains high. Patients should be thoroughly evaluated and informed about these risks before undergoing conversion from VBG. To enhance clinical decision-making, future research should focus on the longitudinal outcomes and rates of complications associated with conversional RYGB and SG procedures post-VBG.
